# Enrichment of Broiler Chickens’ Meat with Dietary Linseed Oil and Lysine Mixtures: Influence on Nutritional Value, Carcass Characteristics and Oxidative Stress Biomarkers

**DOI:** 10.3390/foods10030618

**Published:** 2021-03-14

**Authors:** Sabry M. El-Bahr, Saad Shousha, Mohamed A. Alfattah, Saad Al-Sultan, Wasseem Khattab, Islam I. Sabeq, Omar Ahmed-Farid, Osama El-Garhy, Khalid A. Albusadah, Sameer Alhojaily, Ahmed Shehab

**Affiliations:** 1 Department of Biomedical Sciences, College of Veterinary Medicine, King Faisal University, Al-Ahsa 31982, Saudi Arabia; smmohamed@kfu.edu.sa (S.S.); kbusadah@kfu.edu.sa (K.A.A.); salhojaily@kfu.edu.sa (S.A.); 2 Department of Biochemistry, Faculty of Veterinary Medicine, Alexandria University, Alexandria 21523, Egypt; 3 Department of Physiology, Faculty of Veterinary Medicine, Benha University, Benha 13736, Egypt; 4 Camel Research Center, King Faisal University, Al-Ahsa 31982, Saudi Arabia; malfattah@kfu.edu.sa; 5 Department of Public Health, College of Veterinary Medicine, King Faisal University, Al-Ahsa 31982, Saudi Arabia; salsultan@kfu.edu.sa; 6 Department of Nutrition and Clinical Nutrition, Faculty of Veterinary Medicine, Benha University, Benha 13736, Egypt; wasim.hassan@fvtm.bu.edu.eg (W.K.); ahmed.shehab@fvtm.bu.edu.eg (A.S.); 7 Department of Food Control and Hygiene, Faculty of Veterinary Medicine Benha University, Benha 13736, Egypt; islam.sabek@fvtm.bu.edu.eg; 8 Department of Physiology, National Organization for Drug Control and Research, Giza 12622, Egypt; ebntaimya@yahoo.com; 9 Department of Animal Production, Faculty of Agriculture, Benha University, Benha 13736, Egypt; osama.elsayed1977@yahoo.com

**Keywords:** functional food, flaxseed, meat quality, antioxidants, broilers

## Abstract

This study aimed to evaluate the effect of four combinations of dietary linseed oil and lysine mixtures on performance, fatty and amino acid profiles, oxidative stress biomarkers, cell energy and meat quality parameters of broiler chickens. One hundred and sixty broiler chicks were allocated into four groups. Birds of groups 1–4 were fed diets containing optimum lysine and 2% of linseed oil, optimum lysine and 4% of linseed oil, high lysine and 2% of linseed oil, and high lysine and 4% of linseed oil, respectively, for a period of 35 days. High linseed oil or lysine levels did not affect the performance of the tested birds, but the high level of dietary linseed oil decreased the concentrations of muscles’ saturated fatty acids (SFA). The highest values of ω-3 polyunsaturated fatty (ω-3 PUFA) and arachidonic acids with lowest levels of monounsaturated fatty (MUFA) were detected in the muscles of birds fed diets containing high linseed oils and/or lysine levels. High linseed oil or lysine levels provided the best essential amino acid profile and improved antioxidant components as well as cell energy, and tenderness and redness of the meat. Conclusively, high dietary lysine and linseed oil combinations improved the nutritional value, antioxidant status and cell energy of broiler chickens’ meat.

## 1. Introduction

Cardiovascular diseases (CVD) have been estimated to be a significant cause of morbidity and mortality worldwide accounting for about a third of all deaths [1]. A healthy diet is amongst the most effective weapons against CVD. Omega-3 fatty acid rich food has been shown to be correlated with a lower incidence of CVD [2]. Enrichment of chicken carcasses with omega-3 fatty acids [3], so-called functional foods, is an important research arena in human and animal nutrition. Advantageously, chicken meat contains high protein and low saturated fat [4] and this is the reason for its nomination as a good functional food. In addition, poultry has the ability to accumulate ω-3 fatty acids in the intramuscular fat of meat and other tissues. The quality of the meat, the fatty and amino acids profile and the levels of various nutrients reflect the components of the rations provided to the birds [5]. Although fish oil has inherently low oxidative stability, which causes a rancid taste and odor in poultry products, it is still being used extensively in poultry diet as a source of ω-3 PUFA [6]. The use of plant-based (ω-3) PUFA, including linseed oil, has been reported to be a valuable alternative to fish oil. This alternative vegetable oil provides optimum eicosapentaenoic acid (EPA, 20:5 n-3) and docosahexaenoic acid (DHA, 22:6 n-3) levels [7]. Linseed oil analysis showed a high content (50–55%) of alpha-linolenic acid (ALA,18:3 n-3) [8]. ALA can be bio-transformed into long chain ω-3 fatty acids such as EPA and DHA [9]. Feeding of linseed oil to broilers provided a meat with a high level of ω-3 fatty acids [10]. Therefore, linseed oil could replace the fish oil diet with limited impact on total ω-3 fatty acids [11]. Excessive growth and meat yields of broilers are important economic concerns for the broiler industry. Therefore, the role of food additives in improving birds’ performance and meat quality has been investigated [12,13,14]. One of these attempts is to add essential amino acids to diet to meet or exceed the National Research Council recommendations [15,16]. Lysine (Lys) is one of the essential amino acid necessary for proper growth performance and muscle development of broiler chickens. It was assessed as the second limiting amino acid after methionine in broiler corn–soybean meal based diets [17]. Lysine is the main essential amino acid [18] for broiler muscle building, especially for the turnover of breast muscle protein where it has a role in modulating protein biosynthesis and breakdown levels [19,20]. Noteworthy, dietary supplementation of either linseed oil or lysine reduced abdominal fat deposition in broilers [20,21]. The current study aimed to evaluate the effect of four combinations of dietary linseed oil and lysine mixtures on performance, fatty and amino acid profiles, oxidative stress biomarkers and meat quality parameters of broiler chickens.

## 2. Materials and Methods

### 2.1. Bird Management and Experimental Design

The Institutional Animal Care and Use Committee Research Ethics of Benha University, Faculty of Veterinary Medicine, Egypt, reviewed and approved all experimental protocols (BUFVTM, 07032020) conducted in this study. One hundred and sixty one-day Ross 308 broiler chicks (mixed-sex; 108 male and 200 female) were obtained from a certified local hatchery (Benha, Egypt). Birds were transported to Poultry Research Farm, Faculty of Agriculture, Benha University, Egypt. The current study had a randomized, factorial design with two factors: lysine hydrochloride (2 levels) x linseed oil (2 levels) for a span of 35 days. Birds were randomly assigned to 20 floor pens (1.25 m^2^) divided into four groups with five replicates of 8 birds in each replicate pen (4 × 5 × 8), each fitted with fresh wood shavings for bedding and supplied with feeder and waterer. Birds of groups 1–4 were fed diets containing optimum lysine and 2% of linseed oil, optimum lysine and 4% of linseed oil, high lysine and 2% of linseed oil, and high lysine and 4% of linseed oil, respectively for a period of 35 days. The environmental temperature remained at 33 °C for the first week and decreased steadily to 25 °C at 21 days of age. The relative humidity was 50–60% and the birds were subjected to 23 h of light a day and 1 h of darkness. Integrated lysine levels were at an optimum level (recommended by the National Research Council (NRC)) and lysine level was 0.25% higher than the optimum. The oil used in the experiment was extracted from linseed in a local plant in Qalyubia, Egypt, under our supervision. Birds within the groups were vaccinated against Newcastle disease virus (Hitchner B1), infectious bursal disease virus (Gumbo L strain) and Newcastle disease virus (La Sota) from drinking water at 7, 14 and 21 days of age, respectively. All nutrient components excluding linseed oil and lysine were formulated to meet or exceed the guidelines of the NRC [15] for meat-type broiler chickens. All birds during the experimental period were fed ad libitum with mash feed and clean water. All diets tested were isonitrogenic and isocaloric as shown in Table 1.

### 2.2. Growth Performance, Carcass Characteristics and Sample Collection

At the end of the experiment, birds were weighed individually to determine the average weight gain (WG) and feed intake (FI). The feed conversion ratio (FCR) was determined as the ratio between the real feed consumed (FI) and the weight gained. At the end of the experiment, 15 birds of each group (three birds per replicate) were selected randomly, weighed, and fasted for 12 h prior to euthanization to reduce contamination of the meat during processing [12]. Immediately after evisceration and deskinning, the weights of carcasses, liver, heart, spleen, gizzard and fat pad, as well as breasts, were captured for individual calculation of different yields and percentages. Carcass yields were calculated as percentages of total weight, while carcass parts and organs were calculated as percentages of carcass weight [22]. In addition, liver and breast muscle sections (pectoralis major) were dissected and frozen in a sealed polythene bag at −80 °C for further laboratory assessment of amino and fatty acid profiles, lipid peroxidation, oxidative stress biomarkers, cell energy parameters and enzymatic antioxidants. The remaining portions of the breast meat were immediately used for estimation of physicochemical attributes.

### 2.3. Determination of Profiles of Fatty and Amino Acids in Breast Muscles

Total breast muscle lipids were extracted using a mixture of chloroform and methanol (2:1; *v*/*v*) solution after 2 min of vortexing and 10 min of centrifugation at 1792 g. The derived supernatant was used for the preparation of fatty acid methyl esters (FAME) using a mixture of methanol/sulfuric acid (95:5) and hexane following the method of esterification described previously [23]. Hexane extract obtained from FAME was used for the quantification of free fatty acids using gas chromatography (GC; Agilent Technologies 7890A) equipped with column SP2330 (30 mm × 0.32 mm× 0.2 μm film thickness; Supelco Analytical, Bellefonte, PA, USA) and flame ionization detector [24]. The FAME peaks were analyzed by comparing the retention times of the standard fatty acid mixture (Cat. No. 24073, Sigma-Aldrich, St. Louis, MO, USA) using the Hewlett-Packard ChemStation software (Agilent Technologies Inc., Wilmington, DE, USA). Values of fatty acids were expressed as μg/g of meat tissue. Breast muscle tissues were homogenized, centrifuged and purified [23] and then derivatized [25]. With some modifications [23], derivatized samples as well as standards for amino acids (Sigma-Aldrich, St. Louis, MO, USA) were injected into HPLC (Agilent HP 1200 Series Apparatus, Santa Clara, CA, USA) mounted with Nova-PakTM column C18 (4 μm, 3.9 μm, 4.6 mm) to distinguish and quantify free amino acids (nmol/g meat) as described earlier [26].

### 2.4. Analysis of Oxidative Stress Biomarkers and Antioxidants in the Liver and Breast Muscle Tissues

HPLC (Agilent HP 1200 Series Apparatus, Santa Clara, CA, USA) was used for the assessment of malondialdehyde (MDA), reduced glutathione (GSH) and 8-hydroxy-deoxyguanosine (8-OHdG) levels in the liver and breast muscles. MDA was estimated using protocols described earlier [27,28]. Briefly, a 10% muscular homogenate (*w*/*v*) and ice-cold 0.1 M Tris-HCl pH 7.4 was developed using an ice-cold homogenizer (Glas-Col, USA). The homogenate was centrifuged for 15 min to remove nucleus and debris at 2000 g at 4 °C. Further, 25 μL of 1,1,3,3 tetra-ethoxy-propane (TEP) was dissolved in 100 mL of water to prepare 1 mM of stock standard MDA solution. The 20 nmol/mL working standard was then prepared by hydrolysis of 1 mL of TEP stock solution in 50 mL of 1% sulfuric acid for 2 h at room temperature, which was again diluted with 1% sulfuric acid to produce a final standard concentration of 1.25 nmol/mL for the analysis of the total MDA. The thiol compounds of oxidized and reduced glutathione (μmol/g tissue) were chromatographically detected in a 30 cm × 3.9 mm C18μ Bondapak column at a flow rate of 1 mL/min and at a wavelength of 190 nm. The mobile phase used was 0.0025 M sodium phosphate buffer, pH 3.5, containing 0.005 M tetrabutylammonium phosphate and 13% methanol. The reference standard for glutathione (oxidized and reduced) was used to validate and quantify samples [28,29,30]. The amounts of 8-hydroxy-2’–deoxyguanosine (8-OHdG) in the liver and breast muscle tissues were determined to assess oxidative stress. The 8-OHDG was separated using C18 reversed phase columns in series (Supelco, 5 pm, I.D. 0.46 × 25 cm), with H_2_O/methanol (85:15 *v*/*v*) set as an eluting solution, and with 50 mM of KH3PO4, pH 5.5, at a flow rate of 0.68 mL/min and a wavelength of 245 nm. The resulting chromatogram was compared to the standard purchased from Sigma-Aldrich to determine the concentration of the sample [31]. Both catalase (CAT) and superoxide dismutase (SOD) activities were determined by the spectrophotometric approach. This protocol was mainly based on the decomposition of H_2_O_2_ by CAT [32], and the amount of SOD enzyme inhibiting the autooxidation of pyrogallol at 2 min intervals [33].

### 2.5. Determination of ATP, ADP and AMP Contents in Liver and Muscle Tissues

Hepatic and muscular ATP, ADP and AMP contents (μg/g tissue) were measured by HPLC. Ultrasphere ODS EC 250 × 4.6 mm column was used for separation at mobile phase. Flow rate was 1.2 mL/min and detection was at a wavelength of 254 nm. Phase A consisted of 0.06 mol/L K_2_HPO_4_ and 0.04 M KH_2_PO_4_ dissolved in deionized water and modified to pH 7.0 with 0.1 M KOH, whereas the mobile phase B consisted of 100% acetonitrile. The chromatograms of ATP, ADP and AMP in the samples were identified by comparison with the standards purchased from Sigma Aldrich [34].

### 2.6. Determination of Meat Quality Parameters

Physicochemical attributes of collected breast meat were assessed as outlined earlier [12,35]. In detail, the final breast meat pH was recorded by direct insertion of a pH-meter (Jenway 3510 pH-meter, Cole-Parmer, Staffordshire, United Kingdom) 24 h postmortem. The water holding capacity was expressed as a drip loss (48 h). The drip loss (48 h) percentage was determined for breast meat cuts of identical sizes (50 ± 5 g) and shapes, which were suspended over a plastic net in an airtight plastic box, stored at 5 °C for 48 h, and then reweighed. Drip loss was measured as the percentage of weight loss compared to the original weight of the meat [36]. Cooking loss percentages were also calculated as stated earlier [36], where the muscle cuts were put separately in thin-walled plastic thermo-tolerant bags in the water bath until the core temperature reached 75 °C, cooled to 5 °C in crushed ice, and reweighed to measure the cooking loss. To estimate thawing loss, the breast meat samples were initially weighed before being held frozen at −18 °C and weighed after thawing at 5 °C for 24 h. Total loss was calculated as the weight difference between frozen and thawed meat. Thawing loss is expressed as a percentage [36]. Cooked breast fillet samples were used for the assessment of the Warner-Bratzler Shear Force (WBSF) using the 3343 Universal Test Device Mono column (Instron, Norwood, MA, USA). The cores removed from the anterior end of each breast fillet were sheared perpendicular to the direction of the fiber. The WBSF was calculated on an average of six samples per group [37]. Chromometer CR-410 (Konica Minolta Sensing INC., Osaka, Japan) was used to determine three-color values including lightness (L *), redness (a *) and yellowness (b *) in the raw breast fillet. These color values were then used for the prediction of color intensity or chromium as (C = (a *^2 + b *^2) ^0.5) and color saturation as (Hue angle (h a) = arctg b */a *). For each group, an average of six measurements was determined.

### 2.7. Statistical Analyses

The obtained data were exposed to two-way Analysis of Variance (ANOVA) as 2 × 2 factorial arrangements (2 levels of linseed oil × 2 levels of lysine). SPSS (version 20; IBM, Chicago, IL, USA) followed by Duncan’s multiple comparison tests were used to compare the differences between dietary treatments. Significant differences were observed at (*p* < 0.05).

## 3. Results

### 3.1. Growth Performance and Carcass Characteristics

Significant interaction between linseed and lysine was evaluated for final weight, weight gain (WG) and feed intake (FI) parameters (*p* < 0.05) (Table 2). Final weight and WG increased significantly in birds fed diets containing combinations of either 4% of linseed oil and high lysine or 2% of linseed oil and optimum lysine compared to that of other groups (Table 2). A combination of 4% of linseed oil and optimum lysine was better than the combination of 2% of linseed oil and high lysine in enhancing the final weight and weight gain of tested birds (Table 2). However, FI decreased significantly (*p* < 0.05) in birds fed diets containing a combination of 2% of linseed oil and a high level of lysine compared to that of birds of other groups (Table 2). Highest FI was observed in birds fed a diet containing a combination of 2% of linseed oil and optimum lysine followed by that of birds fed a diet containing a combination of 4% of linseed oil and high lysine, and that of birds fed a diet containing a combination of 4% of linseed oil and optimum lysine (Table 2). The feed conversion ratio (FCR), carcass yield, breast yield and the weight percentage of liver, spleen, heart and fat pads of all experimental groups remained significantly (*p* > 0.05) unchanged (Table 2). However, the gizzard weight percentage increased significantly (*p* < 0.05) in birds fed diet containing a combination of 4% of linseed oil and high level of lysine compared to that of birds of other groups (Table 2).

### 3.2. Profiles of Fatty and Amino Acids in Breast Muscles

Except for stearic acid, different dietary combinations of linseed and lysine did not significantly (*p* > 0.05) change the concentrations of saturated fatty acids (SFA) in the muscles of birds of all experimental groups (Table 3). The concentration of stearic acid was increased significantly (*p* < 0.05) in the muscle of birds fed diets containing a combination of 4% of linseed oil and a high lysine level compared to that of birds of other groups (Table 3). The concentration of stearic acid was statistically similar in the muscle of birds fed diets containing combinations of either 2% of linseed oil and a high lysine level or 2% of linseed oil and optimum lysine level. The lowest concentration of stearic acid was detected in the muscles of birds fed a diet containing a combination of 4% of linseed oil and optimum lysine level compared to that of birds of other groups (Table 3). The concentration of palmitoleic acid increased significantly in the muscles of birds fed diets containing a combination of optimum and high lysine levels mixed with 4% and 2% of linseed oil, respectively, compared to that of birds fed diets containing combinations of optimum and high lysine levels mixed with 2% and 4% of linseed oil, respectively (Table 3). Dietary 4% of linseed oil induced a significant (*p* < 0.05) decrease in the concentration of oleic acid in the muscle of studied birds when combined with either high or optimum levels of lysine (Table 3). However, this decrease was more pronounced when this level of linseed oil (4%) was combined with high lysine level than that of the optimum (Table 3). Dietary 2% of linseed oil induced a significant (*p* < 0.05) increase in the concentration of oleic acid in the muscle of studied birds when combined with either high or optimum levels of lysine (Table 3). Dietary 4% of linseed oil induced a significant (*p* < 0.05) increase in the concentrations of LNA (C18:3) and DHA (C22:6 n-3) in the muscle of studied birds when combined with either high or optimum levels of lysine compared to that of other groups (Table 3). However, this increase was more evident when this level of linseed oil (4%) was combined with high lysine level than that of the optimum. In this regard, combining a2% of linseed oil and high lysine level was more efficient in increasing the concentrations of LNA (C18:3) and DHA (C22:6 n-3) in the muscle of studied birds than that of a2% of linseed oil and optimum lysine combination (Table 3). High levels of dietary lysine or linseed oil was essential to increase the concentration of LA (C18:2) and arachidonic acid (C20:4 n-6) in the muscle, compared to that of muscles of birds fed diets containing optimum lysine of lower linseed oil levels (Table 3). Higher levels of linseed oil were essential to significantly increase the concentration of EPA (C20:5 n-3) in muscle (*p* < 0.05), irrespective of the levels of lysine (Table 3). The concentrations of ETA in the muscle of all studied birds were unchanged significantly (*p* > 0.05) (Table 3).

The effects of different levels of lysine and linseed oil supplementation (optimum and high) on the amino acid contents of broilers meat are shown in Table 4. Essential amino acid analysis showed that, there was no important interaction between dietary linseed oil and lysine. However, the difference was often attributed to the variations in the levels of either linseed oil or lysine (Table 4). The concentrations of arginine, histidine, phenylalanine and threonine essential amino acids were directly linked to lysine concentrations (Table 4), all of which were higher in broiler groups fed a diet containing high lysine levels irrespective of linseed oil levels. However, a higher level of linseed oil was essential in significantly increasing (*p* < 0.05) the concentrations of these essential amino acids in the muscle of birds fed diets containing an optimum level of lysine (Table 4). The concentration of isoleucine, leucine and methionine increased significantly (*p* < 0.05) in muscles of birds fed diets containing 2% of linseed oil irrespective of the levels of lysine (Table 4). The concentration of lysine increased significantly (*p* < 0.05) in muscle of birds fed diets containing 4% linseed oil particularly when mixed with high lysine (Table 4). High linseed oil level was essential in increasing the concentrations of alanine, asparagine and glutamine in muscle of birds fed diets containing an optimum lysine level (Table 4). However, the level of linseed oil was not essential in increasing the concentrations of these amino acids in muscle of birds fed diets containing high lysine level. The concentrations of glycine, proline, serine and tyrosine amino acids increased significantly (*p* < 0.05) in muscle of birds fed diets containing combinations of optimum lysine and 4% linseed or high lysine and 2% linseed levels (Table 4).

### 3.3. Oxidative Stress Biomarkers and Antioxidants in Liver and Muscle Tissues

Oxidative stress biomarkers concentration and enzymatic antioxidant activities in liver and muscle of birds fed diets supplemented with different proportions of lysine and/or linseed oil are shown in the Table 5. In liver tissue, most biomarkers changed significantly in response to the addition of 2% or 4% of linseed oil. However, no significant changes were generated from the interaction of linseed oil with lysine. Different dietary combinations of linseed and lysine did not significantly (*p* > 0.05) change the activities of CAT in the liver of birds of all experimental groups (Table 5). Combining 2% of linseed oil with either optimum or high lysine levels was significantly (*p* < 0.05) efficient in enhancing the concentrations of SOD activity and GSH concentration in the liver of studied birds with a preference to optimum lysine (Table 5). The combination of 2% of linseed oil with either optimum or high lysine levels was significantly (*p* < 0.05) efficient in reducing the concentrations of GSSG, MDA and 8-OHdG in the liver of studied birds compared to that of other groups (Table 5).

MDA concentration increased significantly (*p* < 0.05) only in muscle of birds fed a diet supplemented with a combination of optimum lysine and 4% linseed oil compared to that of birds of other experimental groups (Table 5). The combination of 2% of linseed oil with either optimum or high lysine levels was significantly (*p* < 0.05) efficient in increasing the concentrations of GSSG in the muscles of studied birds compared to that of other groups which remained comparable (Table 5). The concentrations of 8-OHdG were significantly (*p* < 0.05) increased in muscles of birds fed diets containing combinations either of 4% of linseed oil and optimum lysine or 2% of linseed oil and high lysine compared to other groups, which remained comparable (Table 5). GSH concentration was elevated significantly (*p* < 0.05) in muscles of birds fed diets supplemented with combinations of either optimum lysine and 2% linseed oil or high lysine and 4% linseed oil compared to that of birds fed a diet containing either optimum lysine and 4% linseed oil or high lysine and 2% linseed oil (Table 5).

### 3.4. ATP, ADP and AMP Contents in Liver and Muscle Tissues

ATP, ADP and AMP contents in liver and muscle tissues are shown in Table 6. ATP concentrations increased significantly in the liver of broilers fed diets containing a combination of 2% linseed oil and optimum lysine compared to that of liver of birds of other experimental groups, which remained comparable (Table 6). ADP and AMP concentrations increased significantly in liver of broilers fed diets supplemented with 4% linseed oil irrespective of lysine levels (*p* < 0.05). ATP concentrations increased significantly in muscle of broilers fed diets containing a combination of 4% linseed oil and high lysine compared to that of muscle of birds of other experimental groups, which remained comparable (Table 6). ADP and AMP concentrations were elevated significantly (*p* < 0.05) in muscles of birds fed diets supplemented with combinations of either optimum lysine and 4% linseed oil or high lysine and 2% linseed oil compared to that of other groups, which remained comparable (Table 6).

### 3.5. Meat Quality Parameters

The effect of different dietary linseed oil and lysine mixtures on the quality attributes of broiler meat is shown in Table 7. The values of pH, drip loss (48 h), thawing loss, cooking loss, lightness (L *), redness (a *) and chroma (c) were not changed significantly (*p* > 0.05) in all experimental groups. On the other hand, dietary mixtures containing high lysine level, regardless of the amount of linseed oil, were mostly associated with lower yellowness of broiler meat (b *) compared to other groups, which remained comparable. In addition, the dietary combinations containing high levels of linseed oil (4%), in particular when combined with higher levels of lysine, yielded lower WBSF values (more tender meat). The meat color saturation (Hue angle, h) was significantly (*p* < 0.05) increased in the muscle of birds fed diets containing either 2% of linseed oil and high lysine or 4% of linseed oil and optimum lysine compared to other groups, which remained comparable.

## 4. Discussion

### 4.1. Growth Performance and Carcass Characteristics

In the current study, increasing the linseed oil or lysine level did not affect the values of final weight and weight gain of the tested birds. In addition, regardless of lysine and linseed oil levels, all birds showed similar carcass and breast yields. Similarly, previous studies [38,39,40] did not demonstrate any difference in breast, fillet, or abdominal fat yields due to lysine intake in broiler chickens. In the current study, increased feed intake of broilers fed diets containing 2% and 4% of linseed oil could be responsible for the non-significant effect of lysine supplementation on FCR, growth parameters and carcass characteristics [41]. Regarding the negligible performance and breast meat weight changes, the use of up to 4% of linseed oil in chicken broiler’ diets showed a similar effect [42,43,44]. The current dietary incorporation of high lysine levels did not result in a substantial decrease in the relative weight of fat pads, as indicated in earlier research [20]. Similar to the current findings, a lower abdominal fat content of broilers fed diets containing additional linseed oil has been reported [45,46].

### 4.2. Profiles of Fatty and Amino Acids in Breast Muscles

Poultry breast meat fatty acid composition was found to be more directly correlated with the dietary fatty acid profile than any other studied tissues [47]. In the current study, stearic acid is the only fully saturated fatty acid increased by increasing dietary levels of linseed and/or lysine. An inverse relationship was found between the percentages of stearic acid, total SFA and the amount of dietary lysine in pigs [48]. The addition of linseed oil was associated with a decrease in SFA in chicken meat [47]. Authors attributed this to the inhibiting effect of linseed oil on biosynthesis of the main lipogenic enzymes involved in lipogenesis, acetyl-coA-carboxylase and fatty acid synthase [49,50]. However, previous studies and reviews noted that, in comparison to palmitic acid, increased intake of stearic acid did not appear to have a major impact on overall LDL and HDL cholesterol levels [51,52]. Noteworthy, MUFA including PtA (C16:1) and OA (C18:1) were lowest in birds fed combinations of elevated levels of linseed oil and lysine. The low linseed/optimal lysine dietary mixture yielded similar results but was observed only for palmitoleic concentration. In the current study, an inverse relationship has been observed when the muscle contents of MUFA are compared to that of PUFA. Similar findings have been noted previously after 35 days of linseed supplementation in chicken breast meat [47]. In pork, the concentrations of PtA and OA increased linearly with decreasing dietary lysine level [48]. Current study findings revealed that the broiler group fed elevated dietary levels of linseed and lysine exhibited the highest values of total ω-3 fatty acids (ALN, EPA and DHA). These findings confirmed earlier findings which indicated an enrichment of meat with omega-3 fatty acids as a result of feeding PUFA-rich ingredients to mono-gastric animals such as broiler chickens [10,11,47,53,54,55,56]. In animals, linolenic acid (LNA) [56] is the only ω-3 that must be obtained from the diet since it is not endogenic. Linseed oil is one of the richest plant sources of linolenic acid (50–55%) [57], the biological precursor of long chain fatty acids, such as EPA and DHA [9]. These findings further conservatively indicate that lysine could promote the efficacy of LNA conversion to EPA and DHA in ω-3 metabolic pathways. This proposed mechanism is primarily associated with the increased activity of LNA precursor desaturase, Δ6 and Δ5, which is one of the abundant metabolic criteria observed in poultry [58]. Significant increases in breast DHA due to linseed supplementation but coupled with high levels of methionine were also reported [59]. Previous studies have shown that linseed oil contains substantially less linoleic acid (LA) [45] which has been generated from a dietary mixture of low linseed and optimum lysine in the current study. Nevertheless, the highest omega-6 values of linoleic acid and arachidonic acid associated with dietary mixtures containing high lysine levels may also reveal a boosting effect attributable to high lysine levels. Similar patterns were reported in pigs fed low and high dietary lysine than the optimum one, where LA, LNA and total PUFA percentages rose significantly (*p* ≤ 0.014) with increased dietary lysine levels [48]. Increased linseed oil intake, similarly, resulted in higher de novo ω-3 PUFA synthesis and, as a result, increased total PUFA levels in chicken meat [45,60]. Thus, the direct and indirect (de novo synthesis) contributing influence of ω-3 PUFA enriched diets on the composition of meat fatty acids by the incorporation of dietary fatty acids and by the regulation of the lipogenic enzyme expression must be considered [61]. To our knowledge, no or rare studies have examined the nutritional patterns of high linseed oil and/or lysine supplementation in the amino acid profile of broiler meat. Three patterns can be interpreted from the amino acid profile of the broiler meat developed in this analysis. The predominant trend showed that dietary mixtures containing high levels of either lysine or linseed oil or both increased the muscle content of essential and non-essential amino acids such as arginine, histidine, lysine, phenylalanine, threonine, alanine, asparagine, glutamine, proline and serine. The second pattern showed that dietary mixtures containing high dietary linseed oil (4%), regardless of lysine level, negatively influenced some muscle amino acid values involving isoleucine, leucine and methionine. The third trend was the adverse effect on muscle’s glycine content, which occurred in broilers fed high lysine or high linseed oil dietary mixture. In comparison, the dietary combinations of either high lysine and low linseed oil or high lysine and high linseed provided the best essential amino acid profile than that of the other two mixtures. Although the strongest non-essential amino acid profile was also obtained from broilers fed a high lysine/low linseed oil mixture, the high lysine and high linseed dietary combination profile was not better than that provided by the lysine/high linseed oil mixture. The lower content of phytic acid and cyanogenic glycosides in diets supplied with 2% of linseed oil than those containing 4% of linseed oil may be the explanation for the third amino acid pattern. These components have been shown to be adversely conjugated with lysine, histidine, glycine and arginine, thereby decreasing protein digestibility and hindering proteolytic enzymes [62,63]. However, the superior non-essential amino acid profile obtained from broilers fed an optimum dietary lysine and high linseed oil mixture than that obtained from high lysine and high linseed dietary combinations may not support the third pattern. This may be due to the metabolic interaction between high lysine and high linseed, which may require future research.

### 4.3. Oxidative Stress Biomarkers and Antioxidants in Liver and Muscle Tissues

SOD activity and GSH concentration were enhanced and GSSG, MDA and 8-OHdG concentrations were reduced in liver of birds fed a diet containing mixture of 2% linseed oil and lysine compared to that of other groups. These findings indicated that this combination is of high antioxidant potential. Previous findings [53] reported that MDA measured in the liver supplied with linseed oil was the highest compared to other oil sources. In breast meat, the highest concentrations of MDA and 8-OHdG observed in birds fed 4% dietary linseed oil and optimal lysine mixture may be due to increased total PUFA content, in particular LNA, EPA and DHA. These fatty acids had low competence characteristics for deposition in muscle phospholipids, making them more susceptible to oxidative damage [64], and decreased the antioxidant capacity of animals [65]. These results were consistent with those of previous research on broilers [53,66]. The antioxidant potential was increased in the breast muscle of birds fed diets containing combinations of high lysine concentrations with low and high linseed oil. This increment was considerably greater in muscle of birds fed diets containing combinations of high lysine and high linseed oil. However, previous findings [53] reported that MDA measured in the muscle of broilers supplied with linseed oil was the lowest compared to other oil sources. The underlying metabolic mechanisms whereby lysine potentiates the antioxidant capacity of the muscles, despite the high level of linseed oil, require further investigation.

### 4.4. ATP, ADP and AMP Contents in Liver and Muscle Tissues

Dietary oils are essential ingredients that are mostly used to increase the concentration of broiler energy. High ATP values were estimated in the liver of broilers fed a diet containing low linseed oil (2%) and optimum lysine combination compared to other groups. Therefore, the current study suggested that beta-oxidation of linseed oil may be the predominant metabolic pathway used to provide the ATP necessary for improving the birds’ performance. Enzymatic reactions for elongation and desaturation of LA and LNA for the development of long-chain PUFA, particularly ω-3 fatty acids, may be prevalent at the expense of other pathways in the liver of the other three broiler groups fed high linseed oil and optimum lysine dietary mixture or high lysine dietary mixtures. This is presumably supported by the high contents of birds’ desaturase [58]. Broiler liver is the primary site of de novo fatty acid synthesis and lipid metabolism and could therefore affect the fatty acid composition of other tissues such as muscle based on dietary intake [59]. The breast muscle ATP results suggest that high lysine, along with a high PUFA content in linseed (4%), might have maximized the β-oxidation pathway. This means that both metabolic pathways were induced by linseed and lysine in the current research, which is consistent with previous study [67]. De novo long chain ω-3 fatty acid synthesis was more pronounced when linseed oil was added to the broiler diet 17 days pre-slaughtering [47].

### 4.5. Meat Quality Parameters

Analysis of broiler meat quality revealed that different dietary mixtures of lysine and linseed oil did not affect the overall attributes. The majority of meat quality characteristics, including meat color, tenderness, water holding capacity and other muscle characteristics, are strongly linked with muscle pH [68]. The pH recorded in the current study were of low range, though this did not affect dependent technological attributes including drip loss (48 h), thawing loss and cooking loss, all within the perceived range. Previous work [69] noted that there were no related effects on broilers fed high lysine diet, but only higher water loss rates have been reported. Further, high lysine dietary combinations significantly reduced breast meat yellowness and reduced shear force (more tender), particularly when combined with high dietary linseed oil (4%). Earlier studies have indicated that lysine had a contrasting effect on the b* value of the breast muscle in such a way that the higher concentration of lysine decreased muscle yellowness [69,70]. Nevertheless, the lightness and redness of the breast muscle in the present experiment did not negatively shift with increased yellowness. In contrast, increased lysine and energy in the finished diet increased the WBSF of pig meat [71]. Finally, high Hue angle values were correlated with breast meat from broilers fed dietary combination of high lysine and/or high linseed oil, producing carcasses with a more reddish hue (color). Previous studies integrating broiler diets with different linseed oil levels at 7%, 4% and 3% did not demonstrate any difference in meat sensory consistency parameters [43,56,72].

## 5. Conclusions

No major benefits of dietary lysine supplementation on growth performance, carcass and breast yields were observed. The best amino acid profiles were obtained from the dietary mixture of high lysine and 2% linseed oil. The antioxidant capacity of the breast muscle was improved by combining high concentrations of lysine with 2% and 4% of linseed diet; however, hepatic oxidative stability was not improved. Cell energy indices and the fatty acid profiles suggested that high lysine and/or high linseed oil mixtures in the diet optimized the de novo metabolic pathway for the development of ω-3 PUFA. Dietary high lysine combinations considerably decreased breast meat yellowness, shear force, particularly when combined with a high level of dietary linseed oil, and yielded more reddish-colored meat. High levels of PUFA, particularly omega-3, concomitantly with negligible SFA values derived from broiler meat fed a dietary combination of elevated linseed and lysine levels in the current experiment, introduce a promising technique for producing meat with a higher PUFA content and a favorable fatty acid composition. Such meat may of course have cardio-protective properties as well as beneficial effects against the progression of other diseases.

## Figures and Tables

**Table 1:** Ingredient composition and calculated nutrient analysis of starter, grower and finisher diets.

Ingredients (g/kg)	Starter				Grower				Finisher			
	Groups				Groups				Groups			
	1	2	3	4	1	2	3	4	1	2	3	4
Corn	58.97	51.97	59.24	52	65.87	58.79	66.17	59.19	70.07	62.87	70.33	63.03
Soybean Meal	24.36	24.36	24.49	24.49	18.22	18	18.33	18.33	14.49	14.49	14.59	14.59
Corn gluten meal	5	5	4.27	4.27	3.54	3.54	2.84	2.84	4.45	4.45	3.82	3.82
Linseed oil	2	4	2	4	2	4	2	4	2	4	2	4
Poultry by product meal	4	4	4	4	5	5	5	5	5	5	5	5
Wheat bran	2	7	2	7.24	2	7.3	2	6.98	1	6.2	1	6.3
Vitamin-mineral premix ^1^	0.30	0.30	0.30	0.30	0.30	0.30	0.30	0.30	0.30	0.30	0.30	0.30
Mono calcium phosphate	1.16	1.16	1.16	1.16	1.01	1.01	1.01	1.01	0.85	0.85	0.85	0.85
Limestone ground	1.21	1.21	1.21	1.21	1.07	1.07	1.07	1.07	0.95	0.95	0.95	0.95
L-Lysine HCL	0.28	0.28	0.61	0.61	0.30	0.30	0.59	0.59	0.25	0.25	0.52	0.52
DL-Methionine	0.28	0.28	0.28	0.28	0.26	0.26	0.26	0.26	0.21	0.21	0.21	0.21
Choline chloride	0.10	0.10	0.10	0.10	0.10	0.10	0.10	0.10	0.10	0.10	0.10	0.10
Salt	0.34	0.34	0.34	0.34	0.33	0.33	0.33	0.33	0.31	0.31	0.31	0.31
Nutrient specifications												
ME (Kcal/kg)	3035	3035	3035	3035	3130	3130	3130	3130	3200	3200	3200	3200
Crude protein	21.5	21.5	21.5	21.5	19	19	19	19	18	18	18	18
Calcium	0.92	0.92	0.92	0.92	0.87	0.87	0.87	0.87	0.79	0.79	0.79	0.79
Available Phosphorus	0.45	0.47	0.45	0.47	0.43	0.46	0.43	0.46	0.42	0.43	0.42	0.43
Lysine	1.32	1.32	1.65	1.65	1.19	1.19	1.48	1.48	1.05	1.05	1.31	1.31
Methionine	0.63	0.63	0.63	0.63	0.62	0.62	0.62	0.62	0.56	0.56	0.56	0.56
Methionine + cysteine	0.99	0.99	0.99	0.99	0.89	0.89	0.89	0.89	0.82	0.82	0.82	0.82

Groups 1: birds fed diets containing optimum lysine and 2% linseed oil. Group 2: birds fed diets containing optimum lysine and 4% linseed oil. Group 3: birds fed diets containing high lysine and 2% linseed oil. Group 4: birds fed diets containing high lysine and 4% linseed oil. ^1^ Vitamin and mineral premix supplied for each kg of feeds with: Vitamin A 12,000 IU; vitamin D3 2000 IU; vitamin E 10 mg; vitamin K3 2 mg; vitamin B1 1 mg; vitamin B2 5 mg; vitamin B6 1.5 mg; vitamin B12 0.01 mg; Biotin 0.05 mg; pantothenic acid 10 mg; Nicotinic acid 30 mg; Folic acid 1 mg; zinc 50 mg; Manganese 60 mg; Iron 30 mg; Copper 10 mg; Iodine 1 mg; Selenium 0.01 mg; Cobalt 0.01 mg.

**Table 2:** Effect of dietary linseed oil and lysine mixtures on growth performance and carcass characteristics of broiler chickens.

Parameters	Groups				SEM	*p* Values		
	1	2	3	4				
	Optimum Lys		High Lys					
	2% Lin	4% Lin	2% Lin	4% Lin		Lin	Lys	Lin*Lys
Initial weight (g/bird)	98.19	97.99	97.98	98.00	0.52	0.93	0.93	0.919
Final weight (g/bird)	2001.2 ^a^	1951.2 ^ab^	1855.6 ^b^	2032.2 ^a^	22.12	0.18	0.48	0.025
WG (g/bird)	1903.0 ^a^	1853.2 ^ab^	1757.6 ^b^	1934.2 ^a^	21.74	0.17	0.47	0.023
FI (g/bird)	3023.0 ^a^	2803.7 ^c^	2713.6 ^bc^	2962.3 ^b^	43.79	0.87	0.41	0.020
FCR	1.59	1.51	1.54	1.53	0.01	0.18	0.60	0.300
Carcass Yield (%)	57.98	58.32	57.63	56.68	0.398	0.71	0.23	0.432
Breast Yield (%)	31.00	28.91	29.02	30.56	0.544	0.80	0.89	0.111
Liver (%)	5.17	6.08	5.54	5.73	0.290	0.36	0.99	0.540
Spleen (%)	0.27	0.31	0.38	0.33	0.020	0.93	0.14	0.280
Heart (%)	0.94	0.95	0.82	0.90	0.064	0.73	0.53	0.790
Gizzard (%)	3.15 ^c^	2.62 ^d^	3.81 ^b^	4.27 ^a^	0.145	0.90	0.001	0.011
Fat Pad (%)	2.93	3.67	2.48	3.18	0.193	0.08	0.25	0.962

^a–d^ Means within a row not sharing a common superscript differ significantly with corresponding *p* value. Groups 1: birds fed diets containing optimum lysine and 2% linseed oil. Group 2: birds fed diets containing optimum lysine and 4% linseed oil. Group 3: birds fed diets containing high lysine and 2% linseed oil. Group 4: birds fed diets containing high lysine and 4% linseed oil. Lys: lysine; Lin: linseed; WG: weight gain; FI: feed intake; FCR: feed conversion ratio; SEM: standard error of mean.

**Table 3:** Effect of dietary linseed oil and lysine mixtures on fatty acids profile of breast muscles.

Parameters	Groups				SEM	*p* Values		
	1	2	3	4				
	Optimum Lys		High Lys					
	2% Lin	4% Lin	2% Lin	4% Lin		Lin	Lys	Lin*Lys
SFA								
MA (C14:0)	0.72	0.58	0.71	0.68	0.02	0.21	0.27	0.08
PA (C16:0)	16.97	14.18	17.04	14.86	0.67	0.82	0.78	0.09
SA (C18:0)	7.83 ^b^	6.10 ^c^	7.85 ^b^	8.77 ^a^	0.19	0.01	0.004	0.32
C20:0	1.31	1.02	1.3400	1.3875	0.05	0.10	0.06	0.22
MUFA								
PtA (C16:1)	2.38 ^b^	3.06 ^a^	3.37 ^a^	2.52 ^b^	0.09	0.64	0.24	0.001
OA (C18:1)	25.14 ^a^	19.17 ^abc^	22.07 ^a^	15.45 ^c^	0.82	0.00	0.06	0.85
PUFA								
LA (C18:2)	17.78 ^b^	21.07 ^a^	19.35 ^a^	21.21 ^a^	0.49	0.48	0.40	0.02
LNA (C18:3)	2.28 ^d^	2.90 ^b^	2.50 ^c^	3.08 ^a^	0.02	0.000	0.0001	0.62
ETA (C20:4 n-3)	0.75	0.88	0.72	0.82	0.03	0.05	0.40	0.81
EPA (C20:5 n-3)	0.66 ^c^	0.81 ^a^	0.61 ^c^	0.72 ^b^	0.01	0.00	0.02	0.42
AA (C20:4 n-6)	0.95 ^b^	1.17 ^a^	1.28 ^a^	1.18 ^a^	0.02	0.25	0.005	0.01
DHA (C22:6 n-3)	0.73 ^d^	0.94 ^b^	0.85 ^c^	1.03 ^a^	0.01	0.00	0.0001	0.59
Total SFA	26.82 ^a^	21.84 ^b^	26.93 ^a^	25.70 ^ab^	0.58	0.019	0.11	0.13
Total MUFA	27.52 ^a^	22.23 ^ab^	25.44 ^a^	17.97 ^c^	0.79	0.001	0.07	0.50
Total PUFA	23.15 ^b^	27.75 ^a^	25.31 ^ab^	28.04 ^a^	0.45	0.001	0.19	0.31
Total n-3	4.42 ^b^	5.52 ^a^	4.68 ^b^	5.66 ^a^	0.04	<0.001	0.03	0.46

^a–d^ Means within a row not sharing a common superscript differ significantly with corresponding *p* value. Groups 1: birds fed diets containing optimum lysine and 2% linseed oil. Group 2: birds fed diets containing optimum lysine and 4% linseed oil. Group 3: birds fed diets containing high lysine and 2% linseed oil. Group 4: birds fed diets containing high lysine and 4% linseed oil. Lys: lysine; Lin: linseed; SEM: standard error of mean; SFA: saturated fatty acids; MA (C14:0): myristic acid; PA (C16:0): palmitic acid; SA (C18:0): stearic acid; C20:0: arachidic acid; MUFA: mono-unsaturated fatty acids; PtA (C16:1): palmitoleic acid; OA (C18:1): oleic acid; PUFA: polyunsaturated fatty acid; LA (C18:2): linoleic acid; LNA (C18:3): linolenic acid; ETA (20:4 n-3): eicosatetraenoic acid; EPA (C20:5 n-3): eicosapentaenoic acid; AA (C20:4 n-6): arachidonic acid; DHA (C22:6 n-3): docosahexaenoic acid.

**Table 4:** Effect of dietary linseed oil and lysine mixtures on amino acid profile of breast muscles.

Parameters	Groups				SEM	*p* Values		
	1	2	3	4				
	Optimum Lys		High Lys					
	2% Lin	4% Lin	2% Lin	4% Lin		Lin	Lys	Lin*Lys
Essential amino acids								
Arginine	58.18 ^b^	73.62 ^c^	79.06 ^a^	82.78 ^a^	2.84	0.12	0.02	0.32
Histidine	45.21 ^b^	54.04 ^c^	60.23 ^a^	56.47 ^a^	1.57	0.43	0.02	0.07
Isoleucine	53.71 ^a^	43.66 ^b^	47.88 ^a^	41.97 ^b^	1.50	0.02	0.23	0.50
Leucine	78.13 ^a^	64.77 ^b^	71.85 ^a^	52.76 ^b^	2.55	0.01	0.10	0.59
Lysine	59.20 ^b^	73.23 ^a^	61.35 ^b^	73.82 ^a^	1.90	0.05	0.36	0.15
Methionine	22.76 ^a^	18.66 ^b^	21.14 ^a^	16.34 ^b^	0.47	0.0005	0.06	0.71
Phenylalanine	27.66 ^b^	32.50 ^a^	34.37 ^a^	34.24 ^a^	0.91	0.22	0.04	0.20
Threonine	32.42 ^b^	39.30 ^a^	38.93 ^a^	40.27 ^a^	0.93	0.05	0.07	0.16
Valine	70.07	55.74	63.06	61.14	2.61	0.15	0.88	0.26
Nonessential amino acids								
Alanine	43.49 ^b^	54.17 ^a^	58.42 ^a^	50.75 ^b^	1.10	0.51	0.02	0.001
Asparagine	70.80 ^b^	90.85 ^a^	96.59 ^a^	104.97 ^a^	2.53	0.02	0.00	0.27
Glutamine	105.75 ^d^	132.00 ^b^	146.25 ^a^	122.00 ^c^	5.83	0.93	0.22	0.05
Glycine	40.02 ^b^	46.46 ^a^	50.89 ^a^	37.44 ^b^	1.41	0.24	0.75	0.004
Proline	17.88 ^b^	22.39 ^a^	24.45 ^a^	21.04 ^b^	0.90	0.76	0.17	0.05
Serine	26.40 ^b^	32.17 ^a^	29.94 ^a^	28.70 ^b^	0.80	0.18	0.98	0.05
Tyrosine	28.62 ^c^	38.45 ^b^	44.78 ^a^	30.85 ^c^	1.13	0.38	0.08	0.0002

^a–d^ Means within a row not sharing a common superscript differ significantly with corresponding *p* value. Groups 1: birds fed diets containing optimum lysine and 2% linseed oil. Group 2: birds fed diets containing optimum lysine and 4% linseed oil. Group 3: birds fed diets containing high lysine and 2% linseed oil. Group 4: birds fed diets containing high lysine and 4% linseed oil. Lys: lysine; Lin: linseed; WG: weight gain; FI: feed intake; FCR: feed conversion ratio; SEM: standard error of mean.

**Table 5:** Effect of dietary linseed oil and lysine mixtures on oxidative stress biomarkers and antioxidants.

Parameters	Groups				SEM	*p* Values		
	1	2	3	4				
	Optimum Lys		High Lys					
	2% Lin	4% Lin	2% Lin	4% Lin		Lin	Lys	Lin*Lys
Liver								
CAT (nM/g)	13.55	11.85	11.24	11.92	0.32	0.44	0.11	0.09
SOD (nM/g)	68.16 ^a^	52.62 ^c^	59.40 ^b^	49.08 ^c^	1.04	0.000046	0.01	0.23
MDA (nM/g)	28.93 ^b^	37.26 ^a^	30.58 ^b^	41.21 ^a^	0.93	0.000265	0.16	0.55
8-OHdG (nM/g)	180.25 ^b^	223.25 ^a^	188.50 ^b^	224.00 ^a^	2.51	0.000005	0.39	0.47
GSH (nM/g)	4.23 ^a^	3.38 ^c^	3.80 ^b^	3.34 ^c^	0.11	0.01	0.32	0.40
GSSG (nM/g)	0.22 ^b^	0.26 ^a^	0.22 ^b^	0.27 ^a^	0.01	0.04	0.02	0.10
Muscle								
MDA (nM/g)	25.62 ^b^	35.87 ^a^	26.91 ^b^	25.65 ^b^	1.09	0.061	0.063	0.021
8-OHdG (nM/g)	158.91 ^c^	202.26 ^a^	183.24 ^b^	149.62 ^c^	2.33	0.317	0.010	0.000
GSH (nM/g)	3.28 ^a^	2.95 ^b^	2.85 ^b^	3.48 ^a^	0.08	0.386	0.734	0.014
GSSG (nM/g)	0.25 ^a^	0.21 ^b^	0.23 ^a^	0.19 ^b^	0.01	0.007	0.070	0.910

^a–c^ Means within a row not sharing a common superscript differ significantly with corresponding *p* value. Group 1: birds fed diets containing optimum lysine and 2% linseed oil. Group 2: birds fed diets containing optimum lysine and 4% linseed oil. Group 3: birds fed diets containing high lysine and 2% linseed oil. Group 4: birds fed diets containing high lysine and 4% linseed oil. Lys: lysine; Lin: linseed; SEM: Standard error of mean; CAT: catalase; SOD: superoxide dismutase; nM: nanomole; MDA: malonaldehyde; GSH: reduced glutathione; GSSG: oxidized glutathione; 8-OHdG: 8-hydroxy-2’–deoxy-guanosines.

**Table 6:** Effect of dietary linseed oil and lysine mixtures on cell energy.

Parameters	Groups				SEM	*p* Values		
	1	2	3	4				
	Optimum Lys		High Lys					
	2% Lin	4% Lin	2% Lin	4% Lin		Lin	Lys	Lin*Lys
Liver								
ATP (μg/g)	51.36 ^a^	38.19 ^b^	43.08 ^b^	35.62 ^b^	1.28	0.002	0.06	0.28
ADP (μg/g)	15.48 ^b^	19.02 ^a^	17.06 ^b^	20.76 ^a^	0.51	0.004	0.13	0.94
AMP (μg/g)	5.35 ^b^	6.31 ^a^	5.19 ^b^	7.67 ^a^	0.21	0.001	0.18	0.09
Muscle								
ATP (μg/g)	35.43 ^b^	30.75 ^b^	31.54 ^b^	43.98 ^a^	1.26	0.150	0.089	0.005
ADP (μg/g)	14.31 ^b^	17.88 ^a^	16.31 ^a^	13.26 ^b^	0.48	0.791	0.199	0.005
AMP (μg/g)	4.44 ^b^	6.21 ^a^	5.23 ^a^	4.40 ^b^	0.22	0.308	0.270	0.012

^a–c^ Means within a row not sharing a common superscript differ significantly with corresponding *p* value. Group 1: birds fed diets containing optimum lysine and 2% linseed oil. Group 2: birds fed diets containing optimum lysine and 4% linseed oil. Group 3: birds fed diets containing high lysine and 2% linseed oil. Group 4: birds fed diets containing high lysine and 4% linseed oil. Lys: lysine; Lin: linseed; SEM: standard error of mean; ATP: adenosine triphosphate; ADP: adenosine diphosphate; AMP: adenosine monophosphate.

**Table 7:** Effect of dietary linseed oil and lysine mixtures on meat quality parameters.

Parameters	Groups				SEM	*p* Values		
	1	2	3	4				
	Optimum Lys		High Lys					
	2% Lin	4% Lin	2% Lin	4% Lin		Lin	Lys	Lin*Lys
pH	5.57	5.52	5.60	5.71	0.03	0.62	0.10	0.18
Drip loss%	0.97	1.73	1.68	1.54	0.23	0.51	0.59	0.35
Thawing loss %	3.66	4.21	3.02	3.22	0.30	0.54	0.21	0.77
Cooking loss %	17.42	15.65	16.69	15.53	0.55	0.21	0.71	0.79
L* %	54.39	54.56	53.14	55.53	0.47	0.20	0.89	0.26
a* %	12.67	12.29	10.48	11.85	0.31	0.44	0.05	0.18
b* %	13.36 ^a^	13.78 ^a^	11.96 ^b^	12.31 ^b^	0.22	0.40	0.01	0.94
Chroma (c*) %	16.47	15.54	15.45	15.81	0.47	0.77	0.70	0.51
Hue angle (h°)	33.76 ^b^	38.97 ^a^	38.82 ^a^	34.03 ^b^	0.72	0.63	0.70	0.003
WBSF %	3.40 ^a^	2.78 ^b^	2.97 ^ab^	1.91 ^c^	0.18	0.04	0.09	0.54

^a–c^ Means within a row not sharing a common superscript differ significantly with corresponding *p* value. Group 1: birds fed diets containing optimum lysine and 2% linseed oil. Group 2: birds fed diets containing optimum lysine and 4% linseed oil. Group 3: birds fed diets containing high lysine and 2% linseed oil. Group 4: birds fed diets containing high lysine and 4% linseed oil. Lys: lysine; Lin: linseed; SEM: Standard error of mean; L *: lightness; a *: redness; b *: yellowness; c *: chroma; Hue angle (h °): saturation of the meat color; WBSF: Warner-Bratzler Shear Force.
